# Botanical ethnoveterinary therapies used by agro-pastoralists of Fafan zone, Eastern Ethiopia

**DOI:** 10.1186/s12917-017-1149-6

**Published:** 2017-08-09

**Authors:** Teka Feyera, Endalkachew Mekonnen, Befekadu Urga Wakayo, Solomon Assefa

**Affiliations:** 1 Department of Veterinary Clinical Studies, College of Veterinary Medicine, Jigjiga University, Jigjiga, Ethiopia; 2 Department of Basic Sciences, College of Medicine and Health Sciences, Jigjiga University, Jigjiga, Ethiopia; 3 Department of Pharmacology and Clinical Pharmacy, School of Pharmacy, Addis Ababa University, Addis Ababa, Ethiopia

**Keywords:** Ethnoveterinary, Medicinal plants, Livestock diseases, Fafan zone, Agro-pastoralist

## Abstract

**Background:**

In Ethiopia, plant based remedies are still the most important and sometimes the only source of therapeutics in the management of livestock diseases. However, documentation of this indigenous knowledge of therapeutic system still remains at a minimum level. The aim of this study was, thus, to document the traditional knowledge of botanical ethnoveterinary therapies in the agro-pastoral communities of Fafan Zone, Eastern Ethiopia.

**Methods:**

The study employed a cross-sectional participatory survey. Purposive sampling technique was applied to select key respondents with desired knowledge in traditional animal health care system. Data were gathered from a total of 24 (22 males and 2 females) ethnoveterinary practitioners and herbalists using an in-depth-interview complemented with group discussion and field observation.

**Results:**

The current ethnobotanical survey indicated that botanical ethnoveterinary therapies are the mainstay of livestock health care system in the studied communities. A total of 49 medicinal plants belonging to 21 families, which are used by traditional healers and livestock raisers for the treatment of 29 types of livestock ailments/health problems, were identified in the study area. The major plant parts used were leaves (43%) followed by roots (35%). In most cases, traditional plant remedies were prepared by pounding the remedial plant part and mixing it with water at room temperature.

**Conclusion:**

The various types of identified medicinal plants and their application in ethnoveternary practice of Fafan zone agro pastoralists indicate the depth of indigenous knowledge in ethnobotanical therapy. The identified medicinal plants could be potentially useful for future phytochemical and pharmacological studies.

## Background

Livestock production is an integral part of the Ethiopian agricultural sector that approximately shares 40% of the national agricultural output [1]. Previously, it was reported that Ethiopia has the largest livestock population in Africa [2]. However, due to the prevailing animal diseases, the economic benefits gained from this sector still remain marginal. Animal diseases are among the principal causes of poor livestock performance and cause of high economic losses in the country [3, 4].

Conventional veterinary service is still less developed in the country, which is characterized by lack of adequate animal health infrastructure, veterinary clinics, and veterinarians. Furthermore, most modern drugs are expensive and not affordable to the majority of Ethiopian farmers and pastoralists [5, 6]. The majority of livestock raisers in Ethiopia are far away from the sites of animal clinic stations [7]. These factors make Ethiopian livestock raisers rely on endogenous ethnoveterinary knowledge and practices (mainly botanical products) for the management of diseases of their domestic animals. The traditional remedies are socially acceptable, inexpensive and locally available [8, 9].

However, very little of the ethnoveterinary knowledge of Ethiopian famers and pastoralists in relation to the use of medicinal plants is so far properly documented and analyzed [5, 6, 10]. It is estimated that up to 90% of current livestock diseases are managed through the use of traditional medicines [11]. WHO stated: the use of natural products in control of animal and human diseases are considerably effective [12].

In most scenarios, the traditional medical knowledge in Ethiopia is passed verbally from generation to generation. In addition, valuable information can be lost whenever a traditional medical practitioner passes without conveying his/her knowledge on traditional medicinal plants. Similarly, ethnoveterinary practice in the country is being affected by acculturation and depletion of plants as a result of population pressure, drought, environmental degradation, deforestation and over exploitation of the medicinal plants [13, 14]. Consequently, there is a pressing need to document medicinal plants used and the associated indigenous knowledge by conducting ethnobotanical studies [15, 16].

Compared to the multiethnic cultural diversity and the diverse flora of Ethiopia, the studies conducted on the traditional ethnoveterinary medicinal plants in Ethiopia are very limited [17]. In recent years, few ethnoveterniary surveys have been conducted in different areas of the country [10, 17–28]. As it is factual throughout the country, in Ethiopian Somali Regional State (ESRS), ethnoveterinary knowledge is believed to be rich and worth documenting. However, there is gap of information on the level, scope, role and limitations of plant based remedies in the traditional animal healthcare system. Thus, this ethnobotanical survey was initiated in view of documenting the indigenous knowledge associated with utilization of botanical ethnoveterinary therapies for the management of livestock ailments among the agro-pastoralist communities of Fafan Zone, Eastern Ethiopia.

## Methods

### Study area

**Fig. 1:**
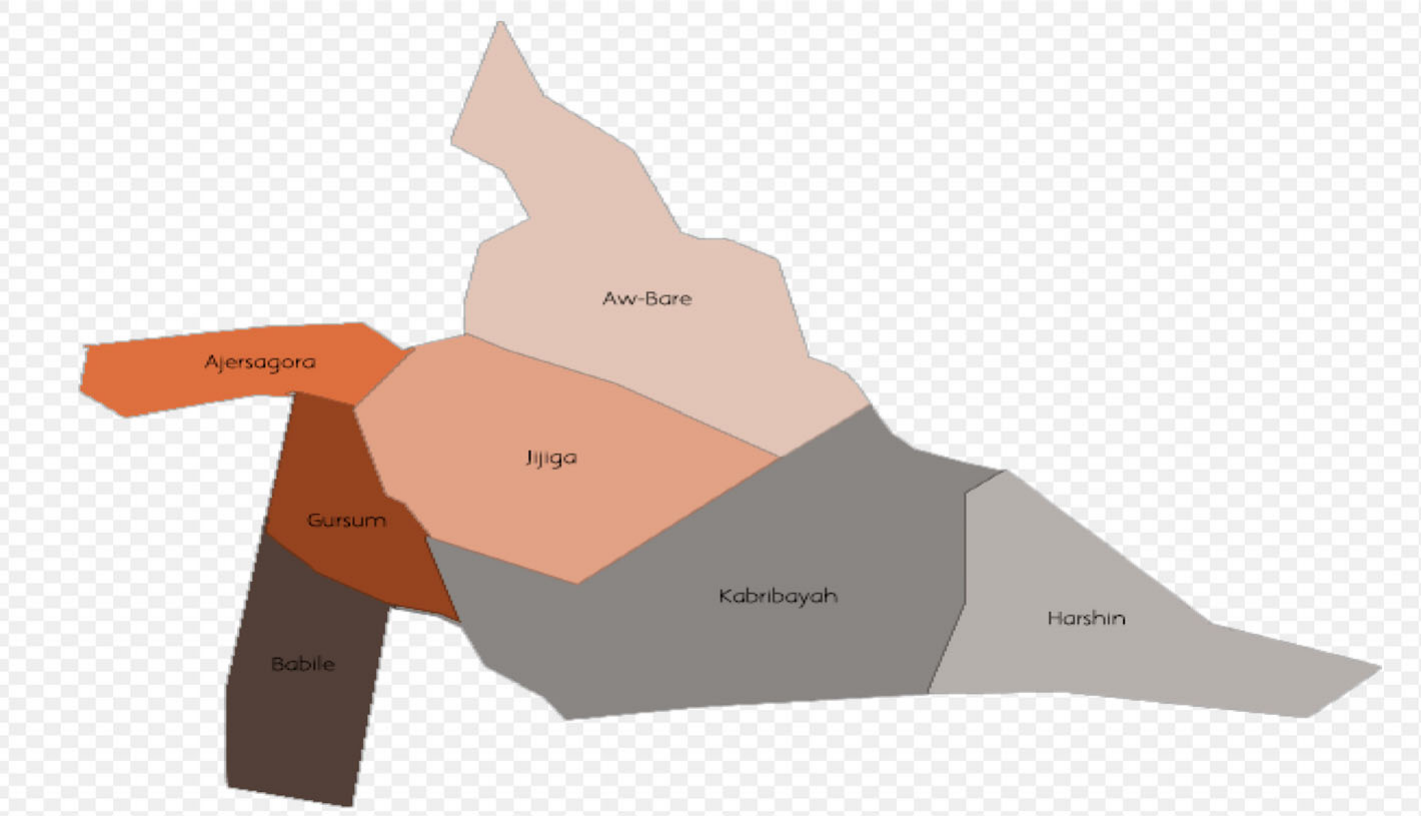
Map of the study area. © User: AlaskaLava / Wikimedia Commons / https://commons.wikimedia.org/wiki/File:Fafan_Zone.png#filelinks / CC-BY-SA-4.0

The study area covers the Babile district and part of Jigjiga district, found in Fafan zone of ESRS (Fig. 1). The zone is situated in the northern part of ESRS. The total land coverage of the zone is 40, 861 km^2^, of which the rangeland extends over 36, 629 km^2^. About 52.6%, 31% and 7% of the landscape of the zone can be categorized as flat to gentle slopes, hills and steep slope, respectively. Fafan zone comprises pastoralism, agro-pastoralism and sedentary production systems. Agropastoralism (95%) is the dominant production system in the zone [29].

The zone geographically lies between 8^°^ 44′ N to 11^°^ 00′ N latitude and 40^°^ 22′ E to 44^°^ 00′ E longitude. The altitude of the zone ranges from 500 to 1650 m above sea level. The mean minimum and maximum temperature ranges from 16 to 20 °C and 28–38 °C, respectively [30]. The rainfall distribution in the zone is very erratic with a mean annual rainfall of 600 to 700 mm [31].

### Study design

A cross-sectional, participatory study was employed to collect ethnoveterinary information from traditional healers in Fafan zone of ESRS between April, 2014 and August, 2015. Indigenous ethno-botanical knowledge, resources and their applications were the main study parameters.

### Sampling procedure

A purposive snowball sampling technique was used to select study participants i.e. ethnopractitioners. This approach aids in acquiring the desired quality and quantity of information on traditional animal health care systems [32]. Ultimately, a total sample of 24 (22 males and 2 female) key respondents were selected.

### Ethnobotanical data collection

Ethnobotanical data were mainly gathered through repeated field trips and investigations, with individual interviews, group discussion, and field observations using the same format used by [33] and [34]. Participant interviews were conducted using semi-structured questionnaires prepared in English and administered in local language (*Somali)* with the help of competent local translators. Data collected comprise: indications, local name, parts used in traditional remedies, mode of preparation (dosage), and route of administration of each medicinal plant against livestock diseases. Moreover, manner of indigenous knowledge transfer was recorded.

### Plant specimen collection and identification

Ensuing interviews with selected key respondents, a field trip was arranged to identify and collect specimen of reported indigenous medicinal plants from their natural vegetation for further botanical identification. Botanical identification of plant specimens was conducted using herbarium materials and taxonomic keys described in various volumes on the Flora of Ethiopia [35, 36]. For each plant species, voucher specimens were given a collection number and deposited in the National Herbarium, Addis Ababa University.

### Enumeration of documented plants

A list of plants and plant products traditionally used to manage animal health problems in the agro-pastoralist communities of Fafan zone was documented. The documentation compiled their scientific and vernacular names, family names, disease and ill-health conditions treated, target type of livestock and the preparation forms of different remedies (Table 2). The names of plants were arranged according to their alphabetical order.

### Data analysis

Microsoft Excel spreadsheet software was employed for organizing and analyzing the collected ethnobotanical data. Descriptive statistical methods (percentage and frequency) were used to summarize data on reported medicinal plants and associated indigenous knowledge.

## Results and discussion

### Socio-demographic characteristics and experience of ethnoveterinary practitioners

Majority of the ethnoveterinary practitioners surveyed in Fafan zone were rural residents and males. Other studies have similarily shown that practice of Traditional Medicine in Ethiopia is largely dominated by men [25, 37]. Majority of the participants have been practicing ethnoveterinary medicine for ≥10 years. Ethno-veterinary knowledge of the traditional healers was usually obtained from family members or religious institutions (Islamic madrasas) which are passed through generation with word of mouth (Table 1). The way traditional veterinary medicine is acquired by the practitioners is largely similar to traditional human medicine. The traditional healers claimed that there is a considerable overlap in the utilization of some of the reported herbs against both human and livestock diseases. It was also interesting to note that most of the sampled ethnoveterinary practitioners were also traditional healers for several human ailments.

**Table 1:** Socio-demographic features and ethnoveterinary experiences of participants (*n* = 24)

Characteristics	Category level	Frequency	Percentage (%)
Sex	Male	22	91
	Female	2	9
Age	25–40	3	12
	41–55	9	38
	56–70	12	50
Residence	Rural	21	88
	Urban	3	12
Educational status	Formal	5	21
	Religious	18	75
	Illiterate	1	4
Level of ethnoveterinary practice experience (years)	< 10	2	9
	10–20	6	25
	21–30	10	41
	>30	6	25
Source of ethnoveterinary healing knowledge	Religious institution	7	29
	Family members or decedents	11	46
	Close friends and colleagues	4	16
	Other senior traditional healers	2	9
Mode of ethnoveterinary service delivery	Always charging	3	12
	Sometimes charging	12	50
	Free (not charging)	9	38

### Documented medicinal plants

The present study showed that the agro-pastoralist communities in Fafan Zone of ESRS use a variety of medicinal plant species to treat a range of livestock health problems. A total of 49 medicinal plants were reported for the treatment of different livestock ailments. The reported medicinal plants are botanically categorized under 21 plant families (Table 2).

**Table 2:** List of traditional medicinal plants used to treat different livestock ailments among the agro-pastoralist communities of Fafan Zone

Scientific name	Family	Vernacular name	Part (s) used	Indication	Method of preparation and application	Livestock species treated	Voucher number
*Abutilon anglosomaliae* Cufod.	Malvaceae	Balanbaal	Leaf	Non-specific external wound	Grounded leaves are applied to wound and washed later	All Livestock	TF-05
*Abutilon bidentatum* Hochst. ex A.Rich.	Malvaceae	Maran	Root	Hyena/Jackal bite wound	Crushed root is applied to affected area	Cattle	TF-25
			Leaf	Helminthiasis, Abdominal pain andSnake bite	Decoction drenched orally	Cattle, sheep and goat	
*Acacia mellifera* (Vahl) Benth.	Mimosaceae	Bilcin	Bark and Root	Retained placenta	Crushed root and bark concocted with *Acacia oerfota* root is administered vaginally to clean uterus	Camel	TF-06
			Bark	Infertility	Bark placed in vagina to kill semen from previous unsuccessful mating	Cattle	
*Acacia oerfota* (Forssk.) Schweinf.	Mimosaceae	Gumar	Bark	Infertility	Bark placed in vagina to kill semen from previous unsuccessful mating	Cattle	TF-34
				Sudden sickness	Bark crushed, mixed with water and drenched orally	Camel	
*Acacia tortilis* (Forssk.) Galasso&Banfi	Mimosaceae	Madheedh	Gum	Non-specific external wound	Gum is applied to wound topically	All Livestock	TF-39
*Adenium aculeatum* (Forsk.)	Apocynaceae	Dhalaandhux	Stem/Root	Ringworm	Crushed root or stem dispersed in water is applied to lesions	Cattle	TF-20
			Stem/Root	Coughing/Pasteurellosis	Decocted and drenched orally	Goat and Sheep	
*Adenium obesum* (Forssk.) Roem. & Schult.	Apocynaceae	Aboobo wan Aad, Aboobo-gunweyn	Stem	Mange infestation	Inside of the stem which has been fermented for two days is applied to mange lesions	Camel	TF-37
*Boscia minimifolia Chiov.*	Capparaceae	Meygaag	Bark and Leaf	Bloat	Crushed bark and leaf mixed with water is drenched orally	Cattle	TF-31
*Carullum speciosa* *N.E.Br* *.*	Asclepiadaceae	Udaabeys	Leaf/Stem	Ringworm	Leaves/stem juice is applied to lesions	Cattle	TF-17
			Leaf	Eye injury or infection	Powdered leaves mixed with oil is applied locally as ointment	Cattle, sheep and goat	
*Catha edulis* (Vahl) Forssk. ex Endl.	Celastraceae	Jaad, qat	Leaf	Helminthiaisis/Diarrhoea	Crushed leaves mixed with water is used as oral drench or mixed with feed and fed	Sheep and goat	TF-28
*Celosia polystachina*	Amaranthaceae	laaleys	Leaf	Non-specific external wound	Crushed leaves mixed with oil is applied to wound	Cattle	TF-22
*Cissus quadrangularis* L.	Vitaceae	Gaad	Aerial part	Tick infestation and external wound	Crushed aerial part mixed with water is applied topically	Cattle and Camel	TF-02
			Leaf	Mastitis, Helminthiaisis and Leach infestation	Crushed leaf mixed with water is drenched orally	Cattle and camel	
			Aerial part	Black leg	Decoction drenched orally	Cattle	
*Cistanche phelypae* L. Cout.	Orobanchaceae	Qoodho-dameer	Leaf and root	Trypanosomiasis	Chopped, mixed with water and drenched orally	Camel	TF-08
*Commiphora erlangeriana* Engl.	Bursuraceace	Dhunkaal	Bark	Tick infestation	Bark crushed, mixed with water, left overnight and used as wash	Cattle, camel, sheep and goat	TF-03
*Commiphora erythrea* (Ehrenb.) Engl.	Burseraceae	Xagar	Leaf/Gum	Mange infestation and ring worm	Cooked gum with animal’s urine is applied to the lesion; Leaf and gum burnt and applied to lesion	Camel	TF-14
*Commiphora ogadensis Chiov.*	Burseraceae	Xagar-madow	Gum	Ringworm	Gum mixed with water is applied to the lesions	Cattle (Calf) and camel	TF-11
*Commiphora serrulata* Engl.	Burseraceae	Mukh	Leaf	Orf	Leaf concocted with *C.drangularis* and mixed with animal urine is cooked and applied to the lesions	Sheep and goat	TF-38
*Crabbea velutina* S. Moore	Acanthaceae	Gheg-maanyo	Leaf	Hyena/Jackal wounds	Grounded leaves applied to wound and washed after three days	Donkey	TF-23
*Crotalaria albicaulis Franch.*	Fabaceae	Gabal-daye	Leaf	Trypanosomiasis	Leaf extracted withwater and concocted with leaf of*C.phelypaef* is drenched orally	Camel	TF-12
*Cucumella kelleri* (Cogn.) C.Jeffrey	Cucurbitaceae	Afgub, uneexo	Root	Infertility	Root is inserted into vagina with *Acacia oerfota* to attract bull	Camel	TF-40
*Cucumis prophetarum* **L.**	Cucurbitaceae	Qalfoon-idaad	Root	Infertility	Root inserted into vagina with *A.oerfota* to attract bull	Cattle and Camel	TF-26
			Fruit	Swellings	Fruit is made warm and bandaged to affected area	All livestock	
				Retained placenta	Crushed and used to wash uterus	Cattle, sheep and goat	
*Cucumis pustulatus* Hook. f.	Cucurbitaceae	Qalfoon	Fruit/Seed	Non-specific external wound	Fruit pulp and seed applied to wound	All Livestock	TF-41
*Cyphostemma cyphopetalum* (Fresen.) Desc. ex Wild & R.B.Drumm.	Vitaceae	Carmo, carmo-waraaboz	Root	Non-specific external wound	Crushed root is applied topically as paste	Cattle, camel, sheep and goat	TF-49
*Cyphostemma serpens* (Hochst. ex A.Rich.) Desc.	Vitaceae	Carom	Root	Non-specific external wound	Powder of dried and crushed root is applied	All Livestock	TF-10
*Dichrostachys cinerea *Wight et Arn.	Mimosaceae	Warsamays	Stem	Hyena/Jackal bite wounds	Burned stem is applied to wound	All Livestock	TF-46
*Echidnopsis dammaniana* Sprenger	Asclepiadaceae	Riyo-dararis	Stem	Lice infestation and Snake bite	Crushed stem mixed with water is used as wash; Crushed and applied to affected area	Cattle (Calf)	TF-45
*Entada leptostachya* Harms	Mimosaceae	Gacma-dheere	Root	Coughing	Grounded root mixed with water is given intranasal; or mixed with feed and fed	Goat	TF-09
*Euphorbia hirta* L.	Euphorbiaceae	Caraba-nadh	Latex	Non-specific external wound	Latex/juice is applied to wound	All Livestock	TF-44
*Euphorbia longispina* Chiov.	Euphorbiaceae	Qabo	Latex	Non-specific external wound	Latex is applied to wound	All Livestock	TF-43
*Euphorbia schizacantha* Pax	Euphorbiaceae	Qabo-yare	Whole plant	Non-specific external wound	Whole plant crushed, dried and used as powder. Juice also applied to the affected area	Cattle and camel	TF-42
*Indigofera amorphoides* Jaub. & Spach	Fabaceae	Meydhax-dheere	Root	Tick and Lice infestation	Crushed (broken) root is applied to ticks/lice	Cattle, sheep and goat	TF-18
			Whole plant	Helminthiasis	Decoction drenched orally	Sheep and goat	
*Ipomoea cicatricosa*e L.	Convolvulaceae	Weylo-wad	Root	Joint diseases	Crushed root is applied topically	Cattle	TF-48
*Jatropha spicata* Pax	Euphorbiaceae	Mawe	Root	Non-specific external wound	Crushed root is applied topically to wound	All livestock	TF-15
			Seed	Indigestion (impaction)	Seed decocted and drenched orally	Cattle, sheep and goat	
*Justica generifolia*	Acanthaceae	Buuxiso	Leaf	Non-specific external wound	Crushed leaves is applied to wound	Cattle	TF-32
*Kleinia abyssinica* (A.Rich.) A.Berger,	Asteraceae	Godor-cad	Rhizome	Sexual impotency	Fresh rhizome is given to bulls to enhance libido	Cattle	TF-35
*Lycium shawii* Roem. & Schult.	Convolvulaceae	Surad	Root	Non-specific external wound /thorns	Crushed root applied near to site of embedded thorns	Camel	TF-29
*Moringa borziana* Mattei	Moringaceae	Mawe	Root	Coughing	Crushed root mixed with boiled water is drenched orally	Sheep and goat	TF-21
*Pergularia daemia* (Forssk.) Chiov.	Asclepiadaceae	Gees-riyaad	Leaf	Non-specific external wound	Leaf juice is applied to affected area	Cattle	TF-16
*Psilotrichum gnaphalobryum* (Hochst) Schintz	Amaranthaceae	Booga-dhaye	Leaf	Non-specific external wound	Crushed leaves concocted with *Ipomoea cicatricosa*e is applied to wound	Donkey	TF-47
*Pupalia lappcea L.* Juss.	Amaranthaceae	Maro-boob, dhegmaanyo	Leaf, fruit or root	Retained placenta, painful joints and wound	Juice or paste is applied to lesion or affected area	Cattle, sheep and goat	TF-04
*Salvadora persica* L.	Salvadoraceae	Caday	Root	Non-specific external wound	Crushed root is applied topically	Cattle	TF-27
*Sarcostemma andongense* Hiern	Asclepiadaceae	Xangey-dhurwaa	Leaf	Snake bite	Leaf juice is applied orally	All livestock	TF-30
*Schinus molle* L.	Anacardiace	Mirmiri	Leaf	Tick infestation	Crushed leaves rubbed on to ticks	Cattle and sheep	TF-01
			Leaf	Eye injury/infection	Leaf Juice is applied topically	Cattle and sheep	
			Bark	Helminthiasis	Water extract of the bark is applied orally	Sheep and goat	
*Seddera pedunculata*ae (Balf.f.) Verdc	Convulvolaceae	Nagadh	Whole plant	Dermatophilosis (skin infection)	Crushed whole plant is applied topically	Cattle and camel	TF-33
*Solanium dubium* fresen	Solanaceae	Urudhi, Xunboox	Fruit	Non-specific external wound	Fruit juice is applied topically	Camel	TF-36
*Solanum incanum* L.	Solanaceae	Waniiye, xunboox, kiriiri	Fruit/Leaf	Tick infestation	Fruit/leaf sap concocted with leaf of *Schinusmolle* is applied on tick infested area	Cattle and camel	TF-07
			Seed	infertility	Seed inserted into vagina to attract bull	Cattle	
			Leaf	Ring worm and swollen joints	Crushed parts extracted in water is applied locally	Cattle and camel	
			Fruit	Coughing/pneumonia/mastitis	Fruit sap is applied orally/nasally or locally	Goat	
*Solanum jubae Bitter*	Solanaceae	Kiriiri, xunboox	Seeds, fruit, and root	Joint disease and Snake bite	Powder of dried and crushed parts is applied topically to the affected area	Cattle	TF-24
*Withnia somnifera* (L.) Dunal	Solanaceae	Guryo-fan	Leaf	Urinary abnormalities	Leaf concocted with *Cissusquadrangularis* and drenched orally	Cattle and camel	TF-13
*Zanthoxylum chalybeum* Engl.	Rutaceae	Geed-dixri	Fruit	Helminthiaisis	Powder of Crushed fruit mixed with water is applied orally as drench	Sheep	TF-19

Data from the present study showed that Mimosaceae (5 species), and Solanaceae, Bursuraceace, Asclepiadaceae and Euphorbiaceae (4 species each) took the superior share of the reported plant families, followed by Vitaceae, Amaranthaceae, Cucurbitaceous and Convulvolaceae (3 species each). In agreement with this study, Solanaceae, Bursuraceace and Cucurbitaceous have also been reported to be dominant families in other parts of the country [25, 38–40]. The fact that Solanaceae, Bursuraceace, Mimosaceae, Asclepiadaceous and Euphorbiaceae contributed relatively higher number of medicinal plants might be attributed to better abundance of species in the study area belonging to these families.

### Parts used, mode of preparation and routes of administration

**Fig. 2:**
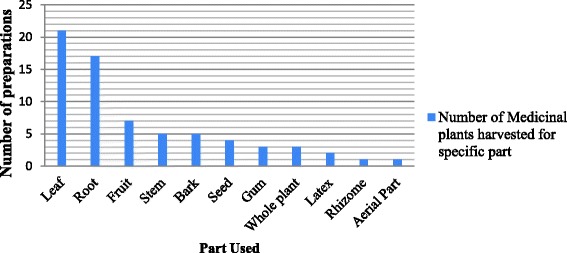
Proportion of plant parts used for preparation of botanical remedies

This study revealed that the most frequently used part of plants was leaf (43%) followed by root (35%) (Fig. 2). Other parts of the plant reported to be used were fruit (14%), stem (10%), bark (10%), seed, gum, latex, rhizome and aerial parts of the plants. Moreover, the entire plant was used in some cases (6%). In consonant with the present study, studies conducted elsewhere in Ethiopia indicated that leaves were the most frequently used plant part to treat livestock ailments [10, 22, 5, 20]. A study conducted by Poffenberger et al. [41] indicated that collection of leaves for traditional remedies poses no significant threat to the survival of plants in comparison with other parts; such as roots, stem, bark and whole plant. On contrary, harvest involving roots, rhizomes, bulb, bark and stem have a serious threat on the survival of the mother plant in its habitat. In this regard, the present study indicated that root was the second commonly utilized part of the medicinal plant, which shows the presence of high risk on the survival of those reported plants in the study area.

In this study, majority (84%) of traditional remedies were prepared using a single medicinal plant. Single plant species based preparations also accounted for majority (65%) of traditional remedies in Afar [5]. However, single plant based preparations were reported at lower frequency from other parts of Ethiopia [22, 42].

In most cases, traditional plant remedies were prepared by pounding the remedial plant part and mixing it with water at room temperature. This is in line with the report of other studies [39, 40]. Some of the plants are prepared and administered in the form of topical route of administration without mixing using water. Topical applications of paste (poultice), sap, and other formulations were reported by other investigators to be common in traditional veterinary practice [18].

### Types of livestock and major livestock health problems treated

**Fig. 3:**
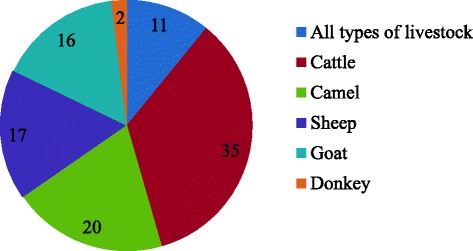
Number of medicinal plants used in different livestock categories in Fafan zone, the area

The therapeutic indication of medicinal plant based remedies in Fafan zone covered all livestock species (Fig. 3) and around 29 distinct disease problems. Medicinal plant remedies were more frequently indicated for diseases affecting cattle and camels, followed by small ruminant and equine diseases. This variation is probably a reflection of the abundance and value of different livestock species in the study area rather than the therapeutic range of medicinal plants themselves.

**Fig. 4:**
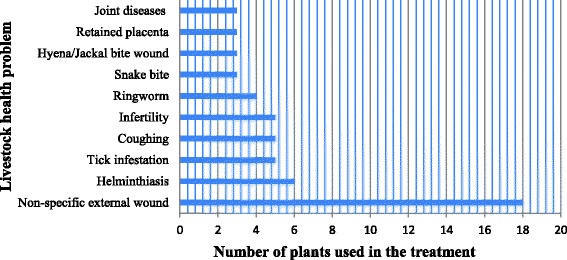
Livestock health Problems against which three or more medicinal plants have been prescribed

Traditional medicinal plant remedies were prescribed against 29 different types of livestock ailments/health problems (Fig. 4). This study generally revealed that most of the traditional medicines used in the area are used for the management of skin diseases and removal of ecto-parasites. Unspecified wounds were reported to be the indication of majority of medicinal plants (18) (Fig. 4), followed by helminthiasis (6), tick infestation, respiratory disorders characterized by coughing and infertility (5). Out of the 29 animal health problems reported to be treated by ethnobotanical remedies, 15 (51.7%) are treated by only one medicinal plant species.

## Conclusions

The study suggests that the agro-pastoralist communities of the study area largely depend on ethnoveterinary medicinal plants for the treatment of different animal ailments. In total, 49 medicinal plants were reported to have been used by the ethnoveterinary practitioners and livestock raisers. Leaf followed by root was the most frequently used plant part in the preparation of ethnobotanical remedies. The identified medicinal plants could be potentially useful for future phytochemical and pharmacological studies. Thus, further studies on biological activity, phytoconstituents and safety profile of the reported medicinal plants is warranted.

## Abbreviation

ESRS: Ethiopian Somali Regional State
